# Nitrogen-doped cyan-emissive carbon quantum dots for fluorescence tetracycline detection and lysosome imaging†

**DOI:** 10.1039/d2ra04945g

**Published:** 2022-11-24

**Authors:** Tongtong Zhu, Lei Cao, Xinyue Kou, Yulu Liu, Wen-Fei Dong, Mingfeng Ge, Li Li

**Affiliations:** School of Biomedical Engineering (Suzhou), Division of Life Sciences and Medicine, University of Science and Technology of China Hefei 230026 P. R. China; CAS Key Laboratory of Biomedical Diagnostics, Suzhou Institute of Biomedical Engineering and Technology, Chinese Academy of Science (CAS) Suzhou 215163 P. R. China gemf@sibet.ac.cn lil@sibet.ac.cn; Chongqing Guoke Medical Technology Development Co., Ltd Chongqing 401122 China; Zhengzhou Institute of Biomedical Engineering and Technology Zhengzhou Henan 450001 China

## Abstract

Tetracyclines (TCs) prevent the growth of peptide chains and the synthesis of proteins, and they are widely used to inhibit Gram-positive and -negative bacteria. For the detection of tetracyclines in cell and *in vitro*, a convenient and simple detection system based on nitrogen-doped cyan carbon quantum dots (C-CQDs) was developed. C-CQDs have excellent excitation-independent properties, the best optimal excitation peak is 360 nm and the best emission peak is 480 nm. Based on the inner filter effect (IFE), the fluorescence intensity of C-CQDs in solution decreases with the increase of tetracyclines. In the range of 0–100 μM, C-CQDs present a good linear relationship with three tetracyclines (CTC, TET, OCT), with *R*^2^ all greater than 0.999. C-CQDs can detect tetracycline in milk samples with recovery in the range of 98.2–103.6%, which demonstrates their potential and broad application in real samples. Furthermore, C-CQDs exhibit excellent lysosomal targeting, as indicated by a Pearson's coefficient of 0.914 and an overlap of 0.985. The internalisation of C-CQDs was mainly affected by lipid raft-mediated endocytosis in endocytic pathway experiments. These experiments indicate that C-CQDs can be effectively used to detect TC content and target lysosomes as an alternative to commercial dyes.

## Abstract 2

Schematic diagram of the synthesis of C-CQDs and experimental results *in vitro* and in cell.

## Graphical abstract

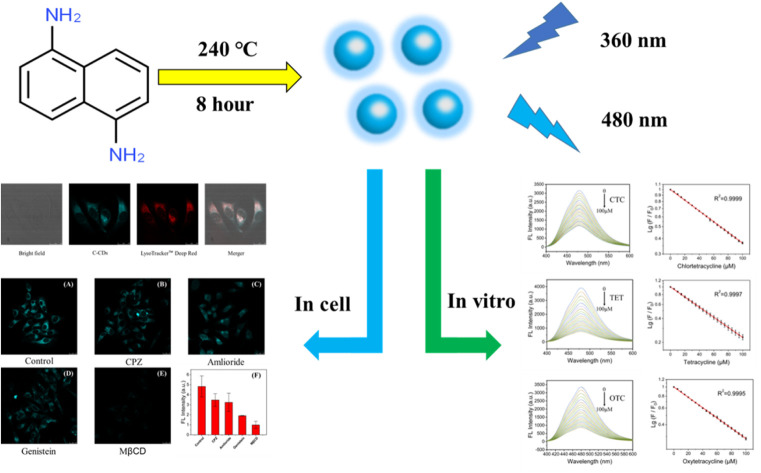

## 1. Introduction

Tetracyclines (TCs) are broad-spectrum antibiotics produced by actinomycetes, and they include natural tetracycline antibiotics such as chlortetracycline, oxytetracycline, doxycycline, and glycyltetracycline.^1^ These antibiotics can specifically bind to the 30S sub-unit of bacterial ribosomes, inhibit the growth of peptide chains, and affect the production of bacterial proteins. Moreover, they have a good inhibitory effect on Gram-positive and -negative bacteria, chlamydia, rickettsia, and other bacteria.^2^ Therefore, they are used to treat human and animal diseases. Tetracyclines are widely used worldwide owing to their low cost, ease of use, and broad-spectrum antimicrobial activity. However, their large use in animal feed can lead to drug residues, which can accumulate in humans and cause gastrointestinal diseases,^3^ nephrotoxicity,^4^ and adverse reactions in the blood or central nervous system.^5^ Furthermore, studies have shown that long-term use of tetracycline can damage teeth by affecting their normal growth and formation.^6^ Thus, the misuse of tetracyclines poses a threat to food security, the environment, and human health. Current methods for tetracycline detection include enzyme-linked immunosorbent assay,^7^ high performance liquid chromatography (HPLC),^8^ capillary electrophoresis,^9^ electrochemical method,^10^ among others. However, these methods require expensive instruments or complicated processes. Therefore, there is a need for a tetracycline detection method with low cost, convenient operation, high sensitivity, and strong linear relationship.

Carbon dots (CDs) are monodispersed spherical and can emit bright fluorescence. Since their first report in 2004, CDs have been receiving significant attention.^11^ Recent studies have investigated their synthesis,^12^ properties,^13^ and applications in depth.^14,15^ Carbon quantum dots have the characteristics of photoluminescence. Most carbon dots have strong absorption peaks in the ultraviolet (UV) region, and some carbon dots can upconvert luminescence. Compared with traditional metal quantum dot materials, CDs have good biocompatibility, excellent water solubility, low cytotoxicity, and low pollution potential.^16,17^ Owing to these excellent properties, CDs have been widely used in biomedical imaging,^18^ phototherapy technology, light-emitting devices,^19^ drug delivery, and many other fields in the past decades.^20^ In particular, CDs-based sensors can be constructed for the detection of substances such as metal ions, anions, biomolecules, and antibiotics.^21^ CDs can be used to detect antibiotics, which has attracted significant research interest.^22,23^ However, the respective detection methods can only identify one tetracycline, and it is difficult to detect multiple tetracyclines while maintaining a high linear relationship. In addition, except for *in vitro* tetracycline detection using CDs, most studies on the utility of CDs in cell only include simple cell imaging. Therefore, further in-depth studies are needed.

**Scheme 1:**
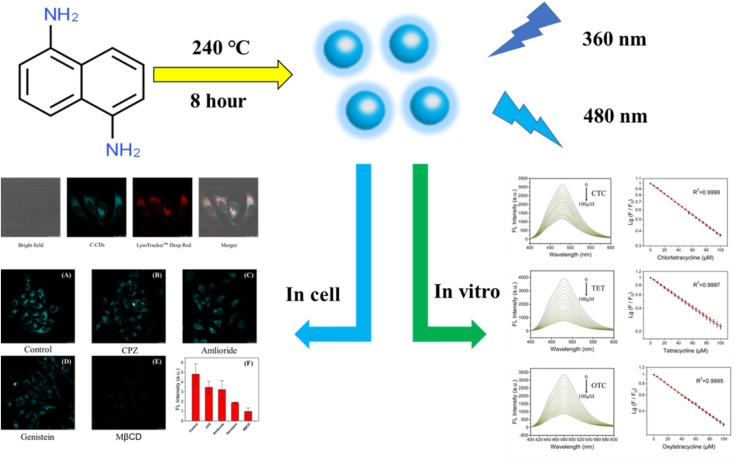
Schematic diagram of the synthesis of C-CQDs and experimental results *in vitro* and in cell.

In this work, cyan carbon quantum dots (C-CQDs) were manufactured by a one-step method using 1,5-diaminonaphthalene (NDA) and concentrated hydrochloric acid. The synthesised C-CQDs presented excellent optical properties and particle size of 7–8 nm. C-CQDs have excellent excitation-independent properties, the best optimal excitation peak is 360 nm and the best emission peaks is 480 nm. The excitation peak of C-CQDs present a high overlap with the UV absorption of tetracyclines, which represents a potential for use in rapid detection of the tetracycline family based on the inner filter effect (IFE). The *in vitro* assay results obtained in this study for chlortetracycline hydrochloride (CTC), tetracycline hydrochloride (TET) and oxytetracycline hydrochloride (OTC) showed that the developed method has a wide detection range (0–100 μM) and strong linear relationship (*R*^2^ > 0.999). Moreover, the cytotoxicity test showed good biocompatibility. The addition of tetracycline to the cells showed that the fluorescence intensity of C-CQDs decreased with the increase of tetracyclines, which indicated a good in cell detection ability. Furthermore, C-CQDs exhibited excellent targeting properties to intracellular lysosomes, and experiments showed that their endocytosis was energy-dependent and inhibited by methyl-β-cyclodextrin (MβCD). Therefore, based on the IFE, C-CQDs can well detect the tetracycline family through changes in fluorescence brightness, which provides a capable tool for tetracycline detection both *in vitro* and in cell (Scheme 1).

## 2. Experimental section

### 2.1 Materials

1,5-Naphthalenediamine (NDA), chlortetracycline hydrochloride (CTC), tetracycline hydrochloride (TET), oxytetracycline hydrochloride (OTC) were purchased from Aladdin Ltd. All chemical reagents can be used directly without additional processing and deionized water is used throughout the experiment.

### 2.2 Instruments

The transmission electron microscopy (TEM) images of C-CQDs were gained by using TECNAI G20 high-resolution transmission electron microscope (FEI, USA). The Dynamic light scattering (DLS) of C-CQDs were determined using a Zetasizer Nano ZS-90 analyzer (Malvern Instruments). The X-ray photoelectron spectroscopy (XPS) spectra were recorded using the Thermo Fisher EscaLab 250Xi X-ray photoelectron spectrometer (Thermo Fisher Scientific, USA). In addition, the Fourier transform infrared (FTIR) spectra were recorded with a VERTEX 70 FT-IR spectrometer (Bruker, Germany). F97Pro FL spectrophotometer (Lengguang Technology, Shanghai) was used to measure the fluorescence spectra of C-CQDs. A UV-vis absorption spectrophotometer (Agilent Cary 300 Scan) was used to detect the UV-vis absorption spectra of C-CQDs. Fluorescence imaging was performed by using an Olympus FV1000 confocal laser scanning microscope (Olympus, Japan). In the DLS test, the concentration of the C-CQDs was 50 μg mL^−1^ with 25 °C. The stability time was set to 2 min, with remaining parameters remained default. In the UV-visible, fluorescence spectroscopy experiments, the C-CQDs concentration was 0.5 mg mL^−1^. If not specifically noted, the experimental temperature was kept at room temperature. The solution of C-CQDs were sonicated for 15 min before the experiments.

### 2.3 Synthesis of the G-CQDs

Using 1,5-diaminonaphthalene (NDA) as a precursor, a one-step solvothermal method was used to prepare C-CQDs. The specific synthesis method is as follows: 0.44 g of NDA was added to 30 mL of absolute ethanol. Subsequently, added 1 mL of concentrated hydrochloric acid. The resulting mixture was hydrothermally treated in a Teflon-lined stainless steel autoclave at 240 °C for 8 hours. After cooling the obtained liquid to normal temperature, rotary evaporation was performed. Deionized water was added to the resulting solid, resulting in a light green liquid. Filter the liquid and perform a second rotary evaporation. Finally, deionized water was added and the supernatant was taken. Then the final carbon quantum dot powder was obtained by a freeze dryer for subsequent experiments. Finally, 0.15 g of C-CQDs were obtained with a yield of 34.1%.

### 2.4 Detection of TCs in real samples

The milk in the experiment was purchased from a local supermarket. After removing protein from milk, cells were centrifuged at 10 000 rpm for 20 min. The supernatant was removed and filtered using a 0.22 μm microwell film to obtain the milk used in the experiments. During the detection process, the C-CQDs prepared on the same day were used, and the concentration of the C-CQDs in the solution was controlled for 0.5 mg mL^−1^. CTC, TET, and OTC at different concentrations (2 μM, 5 μM, 10 μM) were added to detect the change in fluorescence intensity. This experiment was repeated in five times. After 24 hours, the same batch of C-CQDs was taken for the above repeated experiments, still repeated five times.

### 2.5 Cytotoxicity assay

Cytotoxicity was tested using standard WST-1 methods. After placing HeLa cells in a 96-well plates for 24 hours, 0–100 μg mL^−1^ of C-CQDs solution was added to incubate for 24 hours. Then adding 20 μL of WST-1 to the well plate and incubating for 40 min, the absorbance of the solution was measured using microplate reader. The incubation conditions were both 37 °C and 5% CO_2_.

### 2.6 Cellular imaging and endocytosis mechanism study

Taking HeLa as the cell research object. The culture medium consisted of 10% FBS, and 90% DMEM medium. After co-incubation of HeLa cells with Lyso-Tracker™ Deep Red and C-CQDs (60 μg mL^−1^, 30 min), the cells were washed three times with PBS. Inhibitors of four endocytic pathways chlorpromazine (CPZ), methyl-β-cyclodextrin (MβCD), genistein, and amiloride were used in studies investigating the process of C-CQDs entering cells.

## 3. Results and discussion

### 3.1 Characterization of C-CQDs

**Fig. 1:**
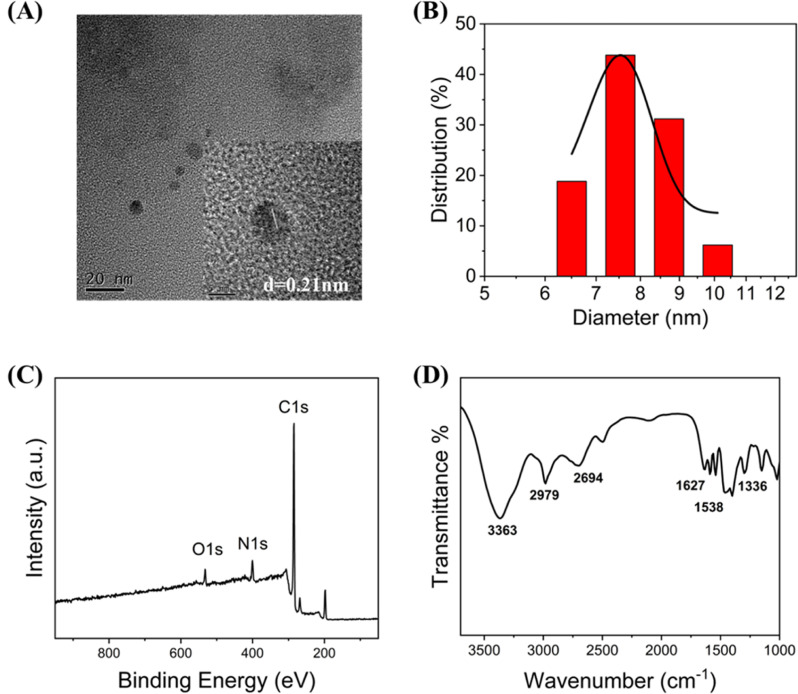
(A) The TEM images of the synthesized C-CQDs in experiments (B) the DLS of the synthesized C-CQDs in experiments (C) the XPS of synthesized C-CQDs in experiments. (D) The FT-IR spectrum of the synthesized C-CQDs in experiments.

Tunnelling electron microscopy (TEM), X-ray photoelectron spectroscopy (XPS), and Fourier transform infrared (FTIR) spectroscopy were used to characterise the C-CQDs. The C-CQDs samples were placed on a copper grid to obtain the tunnelling electron microscopy images. The high-resolution TEM image in Fig. 1A clarified that the C-CQDs are morphologically approximately 8 nm spheres, with a lattice spacing of 0.21 nm. Other size TEM images (Fig. S1†) also show that the particle size of the carbon points is less than 10 nm. In the DLS analysis results, CDs with particle size of 6.5 nm, 7.5 nm, 8.7 nm and 10.1 nm accounted for 18.8%, 43.8%, 31.2% and 6.2%, respectively. It is therefore reasonable to assume that the size of the carbon point is 7–9 nm (Fig. 1B), which meets the requirement of less than 10 nm for CDs and in good agreement with the TEM results. In addition, the XPS measurement results in Fig. 1C showed three clearly distinct peaks at 295.25, 410.65, and 542.80 eV, which correlated with C 1s, N 1s, and O 1s, respectively. C-CQDs are composed of three elements, C, N, and O, with contents of 80.78%, 11.60%, and 7.62%, respectively. Furthermore, two peaks at 284.76 and 286.03 eV in high-resolution C 1s spectrum (Fig. S2A†) corresponded to C=C and C=O bonds, respectively.^24,25^ The high-resolution XPS spectrum of N 1s (Fig. S2B†) showed two characteristic peaks at 399.13 (pyridinic N^26^) and 401.42 eV (amino N^27^). Moreover, the high-resolution O 1s (Fig. S2C†) displayed two main peaks. C=O corresponded to 531.73 eV and C–OH corresponded to 532.58 eV.^28^ The FTIR measurement results revealed the characteristic peaks of C-CQDs. The FTIR spectrum of the C-CQDs is shown in Fig. 1D, and it indicates the presence of –NH_2_ (3363 cm^−1^), C–H (2979 cm^−1^), C–OH (2694 cm^−1^), –C=O (1627 cm^−1^), –NO_2_ (1538 cm^−1^), and C–N–C (1336 cm^−1^). The FTIR results were consistent with the XPS analysis, which demonstrated that there were a large number of amino groups on C-CQDs. These results strongly support the water-solubility and lysosome-targeting properties of C-CQDs.^29,30^

### 3.2 Optical properties of the C-CQDs

**Fig. 2:**
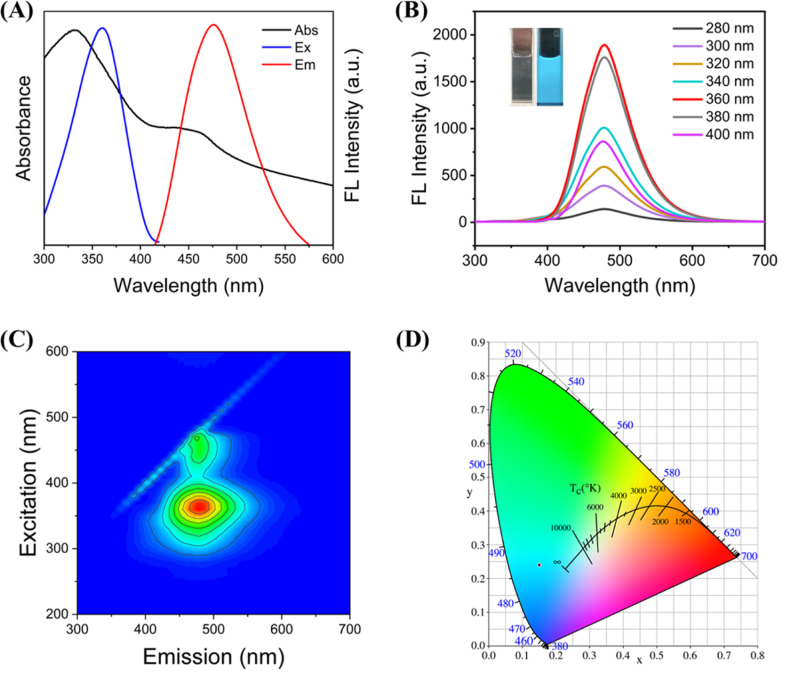
(A) UV-vis absorption, excitation and emission spectra of synthesized C-CQDs. (B) Fluorescence emission spectra of C-CQDs under 280–400 nm. (Inset: images of the synthesized C-CQDs under natural light and UV light, respectively). (C) Total luminescence spectrum of the synthesized C-CQDs, the wavelength is in the range of 200–900 nm. (D) The CIE of C-CQDs.

In addition to the characterization of C-CQDs, optical properties are also an important research aspect of carbon quantum dots. UV-vis absorption, excitation and emission spectra of C-CQDs were investigated in Fig. 2A. Similar to the characteristics shared by carbon quantum dots, the C-CQDs presented absorption bands near 330 nm in the UV-vis spectrum, which was due to the n–π* transition of the surface state.^31^ In addition, the excitation wavelength that provided the best results was 360 nm, and the maximum emission wavelength of C-CQDs irradiated by the optimal excitation wavelength was 480 nm. Tetracyclines also have a strong absorption peak at 360 nm, which provides strong support for the detection based on the IFE principle.

The effects of different excitation wavelengths on the emission peak of the C-CQDs were also investigated in Fig. 2B. The C-CQDs were irradiated with excitation light at 280–400 nm, while the emission peak remained unchanged at 480 nm. Images of the synthesized C-CQDs under natural light was nearly colourless, but can exhibited strong cyan fluorescence under UV light (*λ* = 365 nm). Moreover, the full-scan spectra in Fig. 2C also strongly supported the excitation-independent properties of C-CQDs. The Commission International de l’Eclairage (CIE) coordinates (0.15, 0.24), shown in Fig. 2D, also demonstrate these results. These excitation-independent properties ensure a stable fluorescence emission and the excellent optical properties of C-CQDs, which lay the foundation for subsequent fluorescence imaging in cells.

### 3.3 Stability of C-CQDs

**Fig. 3:**
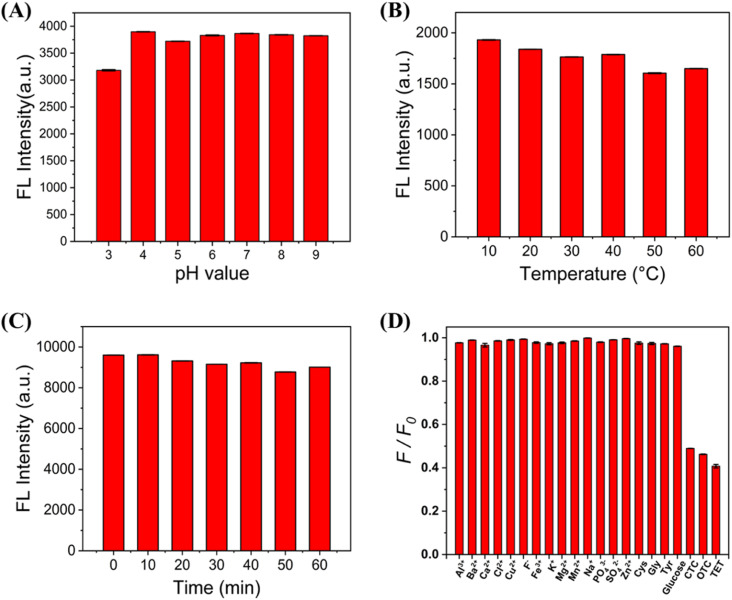
(A) The fluorescence of C-CQDs in aqueous solutions with different pH, to be tested when the solution pH is stable. (B) Changes in C-CQDs when the temperature changed from 10 °C to 60 °C. (C) Changes in the fluorescence intensity of C-CQDs after exposure to UV light for different times. The excitation light wavelength was 365 nm. (D) The ratio of fluorescence intensity of C-CQDs solution before and after adding every substances. The concentration of C-CQDs was 0.5 mg mL^−1^ and all experiments were performed in triplicate.

The temperature, pH and UV irradiation time of the C-CQDs solution may have an impact on C-CQDs, so it is necessary to explore the interference of these factors by means of the fluorescence intensity. The pH of most cells was between 7.35–7.45, and the temperature was 37 °C. Based on these conditions, the fluorescence intensity of C-CQDs under different environments was explored. In Fig. 3A, when the pH changed within the range of 3–9, the fluorescence intensity of C-CQDs did not change substantially and remained at a high level. Moreover, when the solution temperature increased from 10 to 60 °C in Fig. 3B, the C-CQDs also showed good stability. The C-CQDs solution was placed under UV light for different times to detect the fluorescence intensity in Fig. 3C, and there was no clear difference, which indicated good photostability and resistance from external disturbances.

Selectivity is crucial for the detection system of carbon quantum dots. An ideal detection system should detect only the desired substance and not respond to other ions or small molecules. The excitation light in the experiments was set to 360 nm, and a fluorescence intensity of carbon dots was detected at 480 nm when different substances were added. Fig. 3D shows that the fluorescence intensity remained stable upon addition of high concentrations of metal ions (Al^3+^, Ba^2+^, Ca^2+^, Cl^−^, Cu^2+^, F^−^, Fe^3+^, K^+^, Mg^2+^, Mn^2+^, Na^+^, PO_4_^3−^, SO_4_^2−^, and Zn^2+^) some amino acids, and biothiols. In contrast, when low concentrations of the three tetracyclines (CTC, TET, OTC) were added, the fluorescence intensity of C-CQDs decreased significantly. Therefore, the results indicate that the sensor has excellent selectivity and specificity for TCs compared to other substances.

### 3.4 Detection of TCs by the C-CQDs

**Fig. 4:**
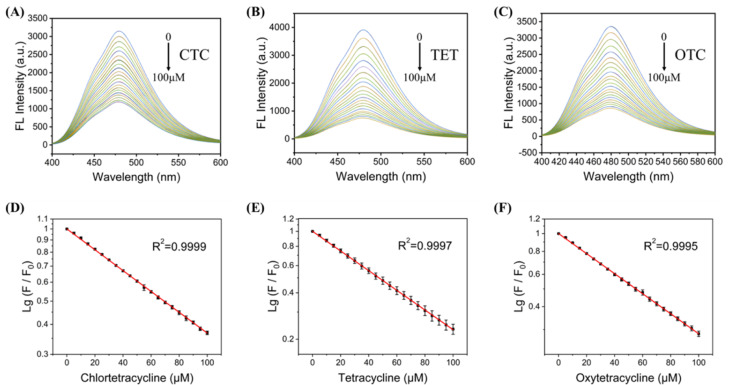
(A) Emission spectra of C-CQDs under excitation light of 360 nm when different concentrations of CTC were added; (B) emission spectra of C-CQDs under excitation light of 360 nm when different concentrations of TET were added; (C) emission spectra of C-CQDs under excitation light of 360 nm when different concentrations of OTC were added; (D) fluorescence linear relationship diagram of C-CQDs when CTC concentration range is 0–100 μM; (E) fluorescence linear relationship diagram of C-CQDs when TET concentration range is 0–100 μM; (F) fluorescence linear relationship diagram of C-CQDs when OTC concentration range is 0–100 μM. The C-CQDs were performed at a concentration of 0.5 mg mL^−1^ and were rapidly detected after the addition of tetracycline to minimize the experimental error. All the experiments were performed in triplicate.

To quantitatively explore the sensitivity of the C-CQDs detection system, three tetracyclines (CTC, TET, OTC) were added to the detection system. The C-CQDs solution was diluted with deionised water to a concentration of 0.5 mg mL^−1^, and then quickly mixed with tetracycline solutions at different concentrations to obtain a C-CQDs-TCs detection system. As shown in Fig. 4A, in the range of 0–100 μM, the fluorescence quenching intensity and CTC concentration showed a excellent linear relationship. The linear regression equation was lg(*F*_0_/*F*) = −0.00633*x* + 0.952 (*R*^2^ = 0.9999), where *F*_0_ is the fluorescence intensity of C-CQDs in the absence of tetracyclines, *F* is the fluorescence intensity of C-CQDs in the presence of tetracyclines. Moreover, the LOD (3*σ*/*k*) was 0.15 μM according to IUPAC regulations.^32^ Furthermore, C-CQDs showed good detection effect for TET in Fig. 4B and OTC in Fig. 4C. Surprisingly, there was a amazing linear relationships in the range of 0–100 μM for both TET and OTC. The linear regression equations are lg(*F*_0_/*F*) = −0.00754*x* + 0.907 (*R*^2^ = 0.9997) and lg(*F*_0_/*F*) = −0.00704*x* + 0.925 (*R*^2^ = 0.9995), respectively. The LOD (3*σ*/*k*) of TET and OTC were 0.18 μM and 0.12 μM, respectively. Therefore, C-CQDs presented a good detection effect on three kinds of tetracyclines (CTC, TET, OTC). Compared with other methods, the detection system based on C-CQDs exhibits the advantages of wide detection range, good linear relationship, and strong real-time detection, with good performance for *in vitro* detection.

### 3.5 Determine the mechanism of TCs

**Fig. 5:**
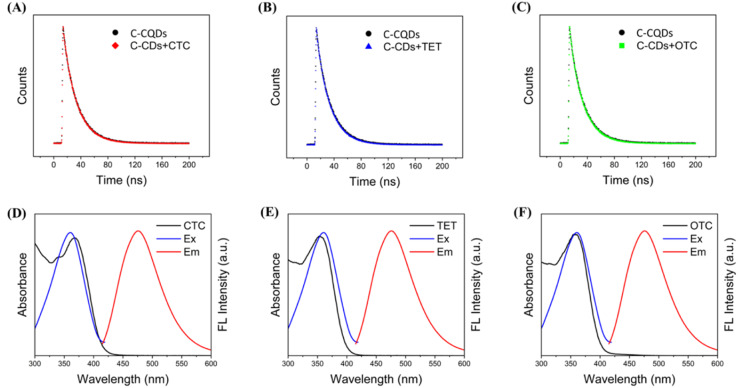
(A–C) Fluorescence decay traces image of C-CQDs, C-CQDs + CTC, C-CQDs + TET, C-CQDs + OTC, respectively. To reduce the experimental error, the absorbance of the solution was less than 0.1. (D–F) UV-vis absorption of CTCs, TET, and OCT, excitation and emission spectra of C-CQDs, respectively. There are highly overlapping regions visible in the figures.

We further investigated the mechanism of C-CQDs detection of tetracyclines. To identify whether the fluorescence quenching was dynamic or static upon addition of tetracycline, the fluorescence lifetime of C-CQDs before and after tetracycline was analysed. As shown in Fig. 5A–C, there was no significant change in the fluorescence lifetime before and after the addition, which indicated that the decrease in fluorescence intensity caused by tetracyclines was static quenching.^33^ Moreover, the UV absorption spectra of tetracyclines were compared with the excitation and emission spectra of C-CQDs, and the results in Fig. 5D–F showed that the UV absorption spectra of the three tetracyclines and the excitation spectra of the C-CQDs presented several overlapping areas. This indicates that in the presence of excitation light at 360 nm, tetracyclines will compete with C-CQDs to absorb excitation light, thereby reducing the fluorescence intensity of C-CQDs. Based on these results, we hypothesised that the fluorescence quenching mechanism between C-CQDs and tetracyclines could be attributed to the IFE.^34^

### 3.6 Detection of TCs in real sample

To explore the applicability of this detection system in real samples, we tested milk samples by the standard addition method using C-CQDs. Tetracyclines were not detected in the milk purchased from local supermarkets. Three concentration gradients of tetracycline were added to the milk samples for detection. As shown in Table 1, the average recoveries of the standard samples were 98.2–103.6%, and the reproducibility (RSD) value were lower than 1.04% (*n* = 5). The inter-day tests showed excellent results, and to measure the stability, the C-CQDs were placed for 24 hours before testing again. As shown in Table S1,† the average recoveries of the standard samples were 97.0–102.1%, and the RSD value were lower than 1.02% (*n* = 5). These results indicated that no significant change in detection effect after placing C-CQDs for one day. Therefore, the inter-day and intra-day tests all showed good results.

**Table 1:** Analytical results for the detection of TCs in real samples (*n* = 5). Milk has been processed and C-CQDs were prepared for the same day and used. To reduce the error, detection was performed immediately after the addition of antibiotics

Tetracycline	Added (μg mL^−1^)	Found (μg mL^−1^)	Recovery (%)	RSD (%)
CTC	2.00	1.98	99.0	0.87
	5.00	5.02	100.4	0.88
	10.00	10.36	103.6	0.94
OTC	2.00	2.05	102.5	0.95
	5.00	5.08	101.6	1.04
	10.00	9.82	98.2	0.98
TET	2.00	1.97	98.5	0.78
	5.00	4.98	99.6	0.90
	10.00	10.08	100.8	0.96

These results show that C-CQDs presented good accuracy, and their use for the detection of TCs in real samples is feasible. Therefore, C-CQDs can quickly and accurately detect tetracycline in real samples, and they have broad application prospects in the field of food testing.

### 3.7 Fluorescence imaging in HeLa cells

C-CQDs showed good detection performance in the *in vitro* experiments for tetracycline detection. In cell experiments, we used C-CQDs as cell imaging probes for tetracycline detection. For that, a standardised WST-1 assay was measured. As shown in Fig. S3,† when the concentration of C-CQDs was 60 μg mL^−1^, the viability of HeLa cells exceeded 90%. These results indicate that C-CQDs have low cytotoxicity and can be used in experiments of HeLa cell imaging.

**Fig. 6:**
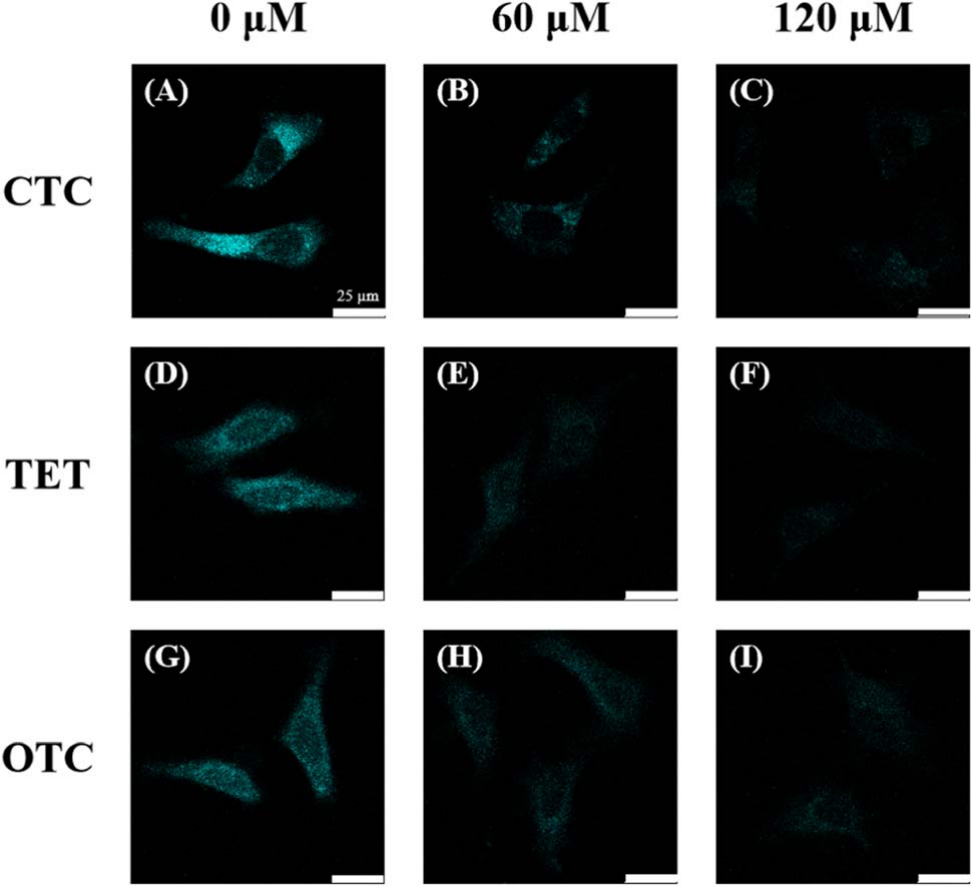
(A–C) Confocal laser scanning microscope (CLSM) images of HeLa cells with the addition of a fixed concentration of C-CQDs and different concentrations of CTC. (D–F) CLSM images of HeLa cells with the addition of a fixed concentration of C-CQDs and different concentrations of TET. (G–I) CLSM images of HeLa cells with the addition of a fixed concentration of C-CQDs and different concentrations of OTC. The concentration of C-CQDs was 60 μg mL^−1^ and the co-incubation is 1 h. The scale bar is 25 μm.

In Fig. 6, after 1 h of co-incubation, C-CQDs clearly entered the cells, exhibited distinct cyan fluorescence, and accumulated mainly in the cytoplasm. In the cells added with 60 μM CTC (Fig. 6B), the fluorescence intensity was significantly reduced by 75%. When the CTC concentration increased to 120 μM (Fig. 6C), the fluorescence intensity decreased to 18%, which was nearly invisible to the naked eye. This indicates that the CTC addition can greatly reduce the fluorescence intensity of C-CQDs in cells, which is consistent with the *in vitro* detection results. Furthermore, similar experimental results were obtained upon addition of the same concentration gradient of TET (Fig. 6D–F) and OTC (Fig. 6G–I). This demonstrated that C-CQDs have good sensitivity to the three tetracyclines. Therefore, C-CQDs can be used for intracellular imaging and detection of tetracyclines in HeLa cells.

### 3.8 Colocalization analysis and mechanism exploration of targeting lysosomes

Mixed elements can bring many functions in carbon point synthesis, such as serving as photocatalyst,^35,36^ enhancing flux recovery ratios,^37^ removing organic dye,^38^ detecting dopamine,^39^ optimizing fuel cell applications^40^ and so on.

In this work, we mainly dope the N element into the carbon dots, and the N element accounts for 11.6%. The addition of N element introduces amino groups, which has been verified in FTIR. A large number of amino groups will make the surface of Cdots positively charged, while lysosomes contain a large number of negative charges, and the mutual attraction of positive and negative charges can provide strong support for C-CQDs targeting lysosomes. It is worth mentioning that, inspired by the excellent research results made by the predecessors, by applying the data of Planck's constant, the speed of light and the UV absorption wavelength (330 nm) of CQDs, the values of the band gap energy (*E*_g_) of the C-CQDs were found to be around 3.76 eV.^41,42^ This result also brings favorable support for C-CQDs targeting lysosomes.

**Fig. 7:**
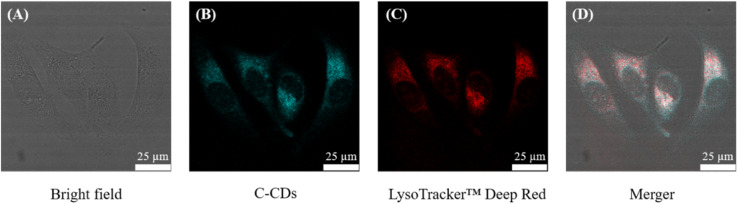
Fluorescence colocalization assay of C-CQDs (A) bright field of HeLa cells. (B) CLSM images of HeLa cells incubated with C-CQDs. (C) CLSM images of HeLa cells incubated with Lyso-Tracker™ Deep Red. (D) Merged by C-CQDs and Lyso-Tracker™ Deep Red. The concentration of C-CQDs was 60 μg mL^−1^. The scale bar is 25 μm.

In subsequent cell imaging experiments, we did observe that C-CQDs always aggregated in the cytoplasm. Confocal images were obtained after the C-CQDs were incubated with the LysoTracker™ Deep Red. As shown in Fig. 7, the cyan fluorescence of C-CQDs largely overlapped the red fluorescence of LysoTracker™ Deep Red. A fluorescence co-localisation analysis was performed using ImageJ software,^43^ and the values of Pearson's coefficient and overlap were 0.914 and 0.985, respectively, which indicated that the C-CQDs indeed aggregated in the lysosomes of cells.

**Fig. 8:**
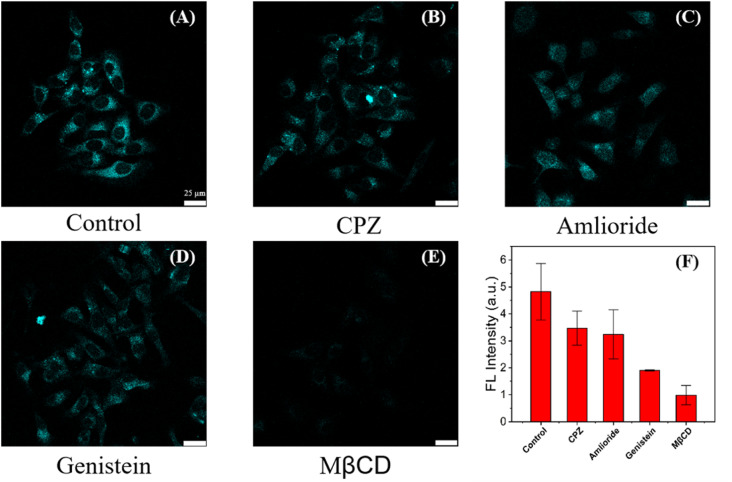
Experiments on the entry of C-CQDs into cells. (A–E) Effects of endocytosis inhibitors (5 mg mL^−1^ CPZ, 10 mg mL^−1^ amiloride, 5 mg mL^−1^ MβCD and 50 mg mL^−1^ genistein for two hour) with 60 mg mL^−1^ C-CQDs, respectively. (F) Average fluorescence intensities of C-CQDs (*n* = 3). The scale bar is 25 μm.

We investigated the potential mechanism of entry into cells of C-CQDs to determine their endocytic pathway. First, the effect of energy supply on cellular uptake was analysed. When HeLa cells were pre-treated at 4 °C for two hour (which stopped the production of adenosine triphosphate and reduced the energy storage in the cells),^44,45^ and other conditions remained unchanged, the fluorescence intensity of C-CQDs in the cells decreased significantly (Fig. S4†), which indicated that the uptake of C-CQDs depends on cellular energy. Second, the effect of different inhibitors on the entry of C-CQDs into cells was investigated.^46^ Chlorpromazine, methyl-β-cyclodextrin (MβCD), genistein, and amiloride, which are inhibitors of clathrin-mediated endocytosis, lipid raft-mediated endocytosis, caveolae-mediated endocytosis and micropinocytosis, respectively, were used as endocytic inhibitors to investigate the pathways of C-CQDs endocytosis.^47^ As shown in Fig. 8, compared with other inhibitors, MβCD presented the strongest effect on C-CQDs. The software analysis showed that after the addition of MβCD, the intracellular fluorescence intensity of C-CQDs decreased by more than 90%, which indicated that the internalisation of C-CQDs was mainly affected by lipid raft-mediated endocytosis. In conclusion, cellular internalization of C-CD is energy-intensive and is significantly affected by lipid raft inhibitors.

## 4. Conclusion

In this study, we prepared nitrogen-doped C-CQDs *via* a one-step method using NDA and concentrated hydrochloric acid. The synthesised C-CQDs presented excellent optical properties and particle size of 7–9 nm. Further experiments showed that C-CQDs presented good stability under different temperature, pH, and ionic environments. In the range of 0–100 μM, C-CQDs presented a good linear relationship with three tetracyclines (CTC, TET, OCT), with *R*^2^ all greater than 0.999. In addition, C-CQDs can detect tetracycline in milk samples with recovery in the range of 98.2–103.6%, which demonstrated their potential and broad application in real samples. Furthermore, fluorescence co-localisation experiments showed that C-CQDs are also useful in targeting lysosomes, with a Pearson's coefficient and overlap of 0.914 and 0.985, respectively. Moreover, the internalisation of C-CQDs was mainly affected by lipid raft-mediated endocytosis by endocytic pathway experiment. In conclusion, C-CQDs can be potentially used to determine the TCs content in foods and as a material for intracellular lysosome imaging.

## Author contributions

Tongtong Zhu: conceptualization, methodology, investigation, writing of the original draft. Lei Cao: conceptualization, methodology, investigation, writing of the original draft. Wen-Fei Dong: methodology, data curation, validation, writing-review & editing. Mingfeng Ge: methodology, data curation, validation, writing-review & editing. Li Li: methodology, data curation, validation, writing-review & editing.

## Conflicts of interest

The authors declare that they have no known competing financial interests or personal relationships that could have appeared to influence the work reported in this paper.

## Supplementary Material

RA-012-D2RA04945G-s001
