# Supplementary material for: Nitrogen-doped cyan-emissive carbon quantum dots for fluorescence tetracycline detection and lysosome imaging
Source: RSC Adv. 2022 Nov 24;12(52):33761–71. doi: 10.1039/d2ra04945g (PMC9685596; doi:10.1039/d2ra04945g)
Supplement: RA-012-D2RA04945G-s001 [file RA-012-D2RA04945G-s001.pdf]

Electronic Supplementary Material (ESI) for RSC Advances.
This journal is © The Royal Society of Chemistry 2022

Supplementary Material

Tongtong Zhu [ab], Lei Cao [ab], Xinyue Kou[ab], Yulu Liu[ab], Wen-Fei Dong [ab], Mingfeng Ge [b*],and Li Li [b*]

a. School of Biomedical Engineering (Suzhou), Division of Life Sciences and Medicine, University of Science and Technology of China, Hefei 230026, P. R. China;

b. CAS Key Laboratory of Biomedical Diagnostics, Suzhou Institute of Biomedical Engineering and Technology, Chinese Academy of Science (CAS), Suzhou 215163, P. R. China;

∗ Corresponding authors.

E-mail addresses: gemf@sibet.ac.cn, lil@sibet.ac.cn.

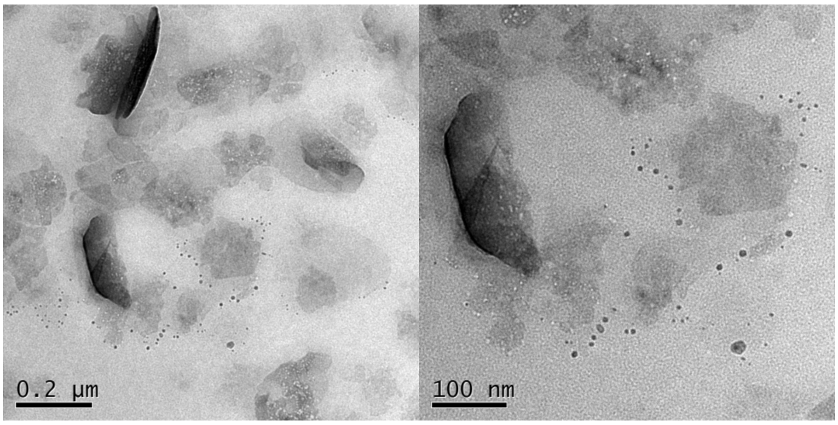

Figure S1. Other size TEM images (0.2 μm,100 nm) of C-CQDs. It is obvious that the size of C-CQDs is mainly distributed below 10 nm, and combining the results of DLS can indicate that the size of C-CQDs is between 7 and 9 nm.

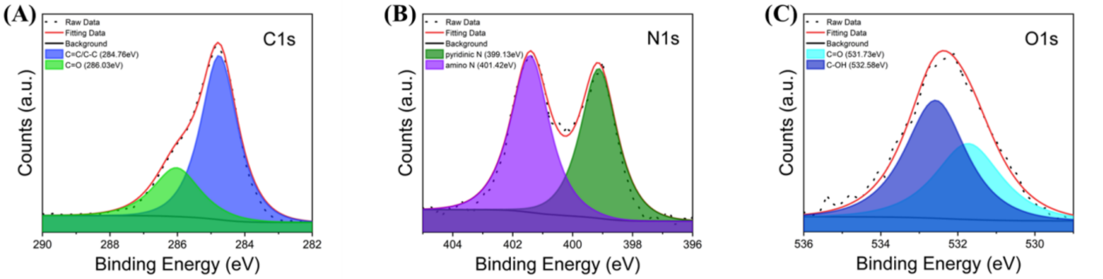

Figure S2. C 1s(A), N 1s(B), O 1s(C) XPS spectrum of C-CDs. The high-resolution C 1s spectrum (A) can be deconvoluted into two peaks at 284.76 and 286.03 eV, which corresponded to C=C/C–C and C=O bonds, respectively. The high-resolution N 1 s spectrum (B) shows two peaks at 399.13 and 401.42 eV, which indicated the presence of pyridinic N and amino N, respectively. The two main bands at 531.73 and 532.58 eV in the high-resolution O 1s spectrum (C) could be identified as C=O and C–OH, respectively.

Table S1. Analytical results for the detection of TCs in real samples (n = 5). Milk has been processed. C-CQDs were prepared one day earlier and used after 24h of placement. To reduce the error, detection was performed immediately after the addition of antibiotics.

Tetracycline	Added (μg/mL)	Found (μg/mL)	Recovery (%)	RSD (%)
CTC	2.00	1.97	98.5	0.88
	5.00	4.99	99.8	0.94
	10.00	10.21	102.1	0.83
OTC	2.00	2.04	100.5	1.02
	5.00	5.04	100.8	0.94
	10.00	9.98	99.8	0.88
TET	2.00	1.94	97.0	0.82
	5.00	4.91	98.2	0.87
	10.00	9.97	99.7	0.97

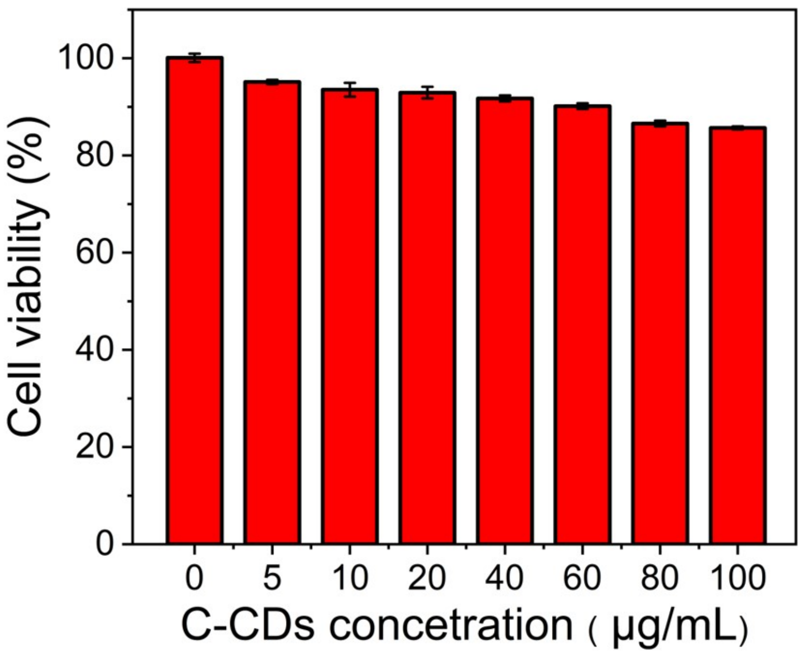

Figure S3. The cell viabilities of HeLa cells under different concentrations of the C-CDs.

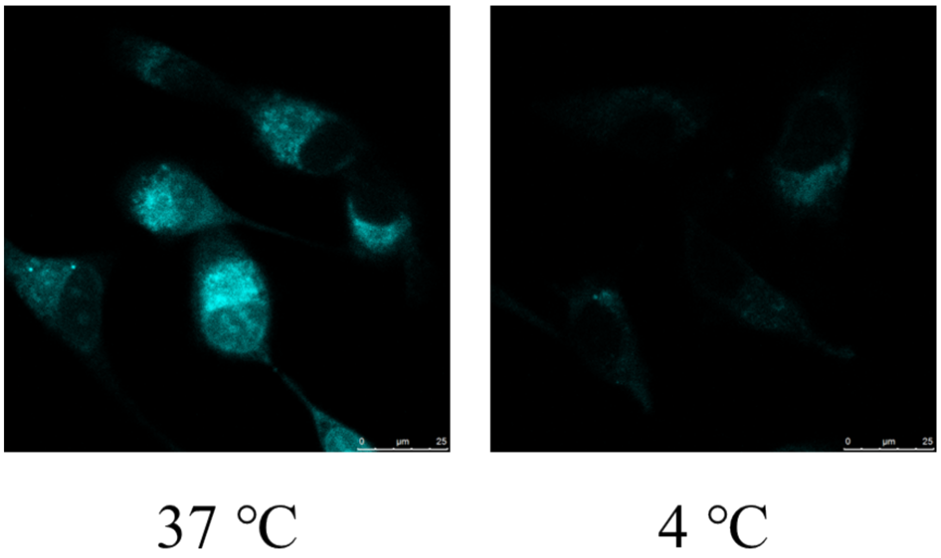

Figure S4. Laser scanning confocal microscope images of HeLa cells under 37°C and 4°C. It can be seen that the fluorescence intensity of C-CQDs in the cells decreased significantly, which indicated that the uptake of C-CQDs depends on cellular energy.
